# TAPS-tool reveals severe under detection of substance use problems in patients with severe mental illness

**DOI:** 10.1371/journal.pone.0305142

**Published:** 2024-07-24

**Authors:** Birgit Seelen-de Lang, Cor de Jong, Giel Hutschemaekers, Robert Didden, Eric Noorthoorn

**Affiliations:** 1 GGZ Oost Brabant, Boekel, The Netherlands; 2 Behavioural Science Institute (BSI), Radboud University, Nijmegen, The Netherlands; 3 GGNet, Warnsveld, The Netherlands; The Ohio State University, UNITED STATES

## Abstract

**Introduction:**

Substance use disorders (SUD) and associated problems are highly prevalent but often undetected in patients with Severe Mental Illness (SMI). This study investigates the prevalence, under-detection, and variables associated with a high risk of SUD in a Dutch sample of adult outpatient SMI patients (*N* = 83).

**Methods:**

Substance use (The Tobacco, Alcohol, Prescription medication, and other Substance use -TAPS-tool), quality of life (Manchester Short Assessment of Quality of Life—MANSA), general functioning (Health of the Nation Outcome Scale–HoNOS), DSM-5 classifications and patient characteristics (age, education, marital status) were assessed. Detection of SUD was determined by calculating % agreement of DSM-5 classification to TAPS outcome. A logistic regression analysis was performed to determine the association of patient characteristics, quality of life and general functioning to an increased risk of SUD as determined by the TAPS.

**Results:**

Concerning prevalence, 89% of the patients used tobacco, above guideline-recommended daily limits of alcohol, illicit drugs or prescription medications for nonmedical purposes. Almost all smokers, half of the alcohol users and three-quarter of the patients that use marihuana or stimulant drugs had a high risk of SUD. All patients with high risk of SUD associated with alcohol, drugs or medications also had SUD associated with tobacco use. Concerning under detection less than half of the patients with a high risk of SUD according the TAPS had a SUD in their DSM-5 classification. Gender, partner, age and satisfaction about the relationship with family had a significant association with a high risk of SUD.

**Conclusions:**

Screening for addiction in an SMI sample with the TAPS-tool revealed a high prevalence of substance use and a high risk of SUD. TAPS outcomes compared to the clinically obtained DSM-5 classification revealed a high degree of under-detection of substance use problems. Smoking seems to pose a specific additional risk of addiction and deserves more attention in treatment to achieve greater health care benefits.

## Introduction

Substance use is common among patients with mental disorders. Substance use disorders (SUD), defined as uncontrolled use of a substance despite harmful consequences, are also common despite leading to problems in social or occupational life. This is especially true for patients with Severe Mental Illness (SMI), such as schizophrenia, bipolar disorder or chronic depressive disorders with psychotic characteristics. Criteria of SMI are met for those in whom the mental disorder is not transient nor in symptomatic remission and accompanied by problems in social and occupational functioning [1, 2]. The prevalence of SMI is estimated between 1.5% and 4% of the population, depending on which definition is used [2–4]. The prevalence of substance use, misuse, and SUDs (including those of prescribed or non-prescribed medication) in people with SMI is twice as high as in those without SMI [5–7]. Rates vary between 30% and 80% depending on whether the studies focus on problematic substance use, substance dependency or SUD [8–10]. Two systematic reviews show a comorbidity of SUD in 33% of the patients with bipolar disorder and in 42% of those with schizophrenia [11, 12]. Over 50% of SMI patients meet criteria for SUD in their lifetime [13]. The point prevalence of SUD for patients with SMI is 25%-30% [14]. Research on addiction in patients with SMI mostly focusses on addictions other than tobacco [11, 12]. In SUD, alcohol is the most commonly used substance, followed by cannabis and stimulant drugs such as cocaine and amphetamine [11, 12, 14].

Biological, psychological and social risk factors make patients with SMI vulnerable to develop SUD. Their brain has higher biochemical sensitivity for psychoactive substances than people in the general population. They experience more negative consequences from using relatively small amounts of substances, are more often exposed to psychoactive substances (they often live in neighborhoods where many people use drugs), and lack social skills to refuse substances. Besides, they experience more stress, have insufficient social support and use substances as self-medication due to a lack of more healthy coping skills [14]. Consequences of SUD in patients with SMI are: more (re)admissions, homelessness, behavioral problems, suicide attempts, death, exacerbation of mental health problems as well as exacerbation of addiction problems, poorer treatment outcome and higher societal costs as result [13, 15, 16].

Often, substance use problems are not recognized in patients with SMI [15]. This has several reasons:

1. Negative (social and functional) consequences of substance use problems may not be visible because of mental health complaints and / or social withdrawal;
2. Cognitive and emotional effects of substances may be mistakenly attributed to symptoms of the psychiatric condition instead of an addiction problem;
3. Mental health staff do not have skills and knowledge to detect substance use problems;
4. Lack of screening and assessment tools which have proven useful in daily mental health practice for people with SMI [8, 14, 17–19]. Available screeners focus only on one substance, and do not incorporate misuse of (prescribed or non-prescribed) medication. Examples of such screeners are: the ‘DAST’ (Drug Abuse Screening Test: [20]), ‘MAST’ (Michigan Alcoholism Screening Test: [21]) and ‘CAGE’ (Cutting down, Annoyance resulting from criticism Guilt feeling, Eye opener: [22]);
5. Assessment instruments, such as the ‘ASI’ (Addiction Severity Index: [23]), need training, are time consuming and need encoding afterwards. Many SMI patients have difficulty concentrating for such a long period. They may have information processing problems, and may be too flustered to take the extensive assessment.

To reduce further adverse consequences of substance use problems, substance use must be identified earlier during treatment for SMI patients with an instrument that is easy to use and screens for multiple substances. In 2016, Wu et al. [24] developed the TAPS tool (Tobacco, Alcohol, Prescription medication and other Substance use/misuse tool) based on the WHO ASSIST-lite. The TAPS-tool is a 2-phase instrument, which consists of a screener (phase 1) to detect patients at risk for unhealthy substance use and an additional short assessment (phase 2) which provides substance-specific risk scores that identify a SUD. Cross-cultural studies have shown promising results pertaining to its feasibility, acceptability and validity [18, 25–27]. Because of its brief nature and screening for various substances, the TAPS-tool seems to be a suitable tool to identify SMI patients with a high risk of SUD.

In this study, we investigated three topics in a sample of adult SMI patients:

1. Prevalence: we investigated the frequency of used substances as measured with the TAPS-tool as well as the proportion of SMI patients with a high risk of SUD.
2. Under-detection: we explored the correspondence between the TAPS-outcomes and the clinical diagnosis of SUD according to the DSM-5.
3. Associations: relationships between patients’ mental and social functioning, their quality of life, patient characteristics (age, gender, level of education, marital and ethnic status) and a high risk of SUD were explored.

## Materials and methods

### Participants and setting

Participants were adult SMI patients aged over 23 years who received treatment in four Flexible Assertive Community Treatment (F-ACT) teams of GGZ Oost Brabant, The Netherlands. This is a secondary mental health trust with several inpatient and outpatient locations in urbanized rural areas in the south-eastern Netherlands. F-ACT is an outreach outpatient program for patients with SMI provided by multidisciplinary teams in the community. The intensity of treatment is flexible and tailored to the patient’s needs at any given time. F-ACT focuses on helping people to function in their own environment [28].

Between September 2020 and February 2023 we collected data from 83 patients who gave written informed consent to use their anonymous data for this study.

### Procedure

Using screening and assessment instruments is care as usual for the participating F-ACT teams to identify the patients’ problems in order to tailor treatment to their needs. Screening and assessment instruments are used at the beginning of treatment and annually thereafer, and of which several (see below) are used in this study. All new incoming patients between September 2020 and February 2023 were asked to give their consent in joining the current study at intake. Treatment in the F-ACT team was the only inclusion criterion. Until the necessary number of participants for the study was reached, case managers asked patients already in care at their annual assessment if they too wanted to participate in the study. There were no more than one measurement in any of the participants. The sample of patients already in care was slightly older, but the two samples did not differ significantly on other patient characteristics (sex, age, level of education, marital status, ethnicity, DSM-5 classification). In this descriptive study, the results of the new incoming patients and the results of the annual assessments were combined and analyzed.

Exclusion criteria for both groups were: incapable of concentrating for more than 15 minutes, Dutch language problems, insufficient cooperation with the F-ACT treatment and mild intellectual disabilities (MID). To detect MID we used the Screener of Intelligence and Learning disabilities (SCIL; [29]) or DSM-5 classification mild intellectual disability. (A version of the TAPS for patients with MID is under development).

All team members received instructions by the team psychologist about how to use and complete the instruments. At the invitation by phone, information was given about the aims of the study and patients were asked if they would like to consider voluntary participation. During the appointment after at least one reflection day, information about the study was reviewed again, an information leaflet was given, and any additional questions from the patients were answered. When the patient agreed to participate, an informed consent form was signed. The study was approved by the institutional scientific board of GGZ Oost Brabant and the Ethics Committee of the Faculty of Social Sciences of the Radboud University at Nijmegen (ECSW-2021-051). The study was registered at ClinicalTrials.gov (Identifier: NCT05273021).

### Measures

From 2020 onwards, the Dutch version of the TAPS-tool was part of standard care in the F-ACT teams of GGZ Oost Brabant. Next to the outcome of the TAPS-tool, mental health symptoms and quality of life were ascertained by using the Health of the Nation Outcome Scale (HoNOS; [30]) and the Manchester Short Assessment of quality of life (MANSA; [31]). Data from the TAPS, HoNOS and MANSA along with patient characteristics such as age, sex, ethnicity, marital status, level of education, and the clinically obtained DSM-5 classification including GAF-score were collected in this study.

### Tobacco, Alcohol, Prescription medication and other Substances-tool (TAPS)

The TAPS-tool is a 2-phase screening and assessment instrument for detecting substance use problems. If the respondent provides a positive answer to any of the past-year substance use questions, (TAPS-1), they are asked brief assessment questions covering the past 3-months (TAPS-2). The TAPS-tool investigates four categories of substances: tobacco, alcohol, drugs (marihuana, stimulant drugs and heroine) and medication (opioids, anxiolytics and stimulant medication). It takes about 10–15 minutes to assess and can be used as an interview or as a self-administered questionnaire with similar results [26]. TAPS-2 questions assess problems associated with each of the substances endorsed in TAPS 1 for the past three months. Item responses on the TAPS-2 are summed per category to calculate substance-specific risk scores to identify a SUD. The scoring options are low risk = 0, moderate risk = 1, and high risk ≥ 2 of a SUD (e.g. alcohol, cannabis, stimulant medication) separately. The TAPS-tool items, skip pattern and scoring system is showed in S1 Fig. Psychometric properties of the TAPS are good in adult primary care settings [18]. If there is any positive score on the TAPS-2, further assessment by additional questions or an assessment instrument is recommended to confirm the SUD diagnosis. For an overview of the development of the TAPS-tool, questions and scoring system, see [18, 24, 26, 32].

### Health of the Nation Outcome Scale (HoNOS)

The HoNOS is a proxy instrument measuring mental and social functioning of a patient in mental health care at a given moment by 12 questions (reflection period: past two weeks). A trained professional can complete the questionnaire in an interview with a patient. The questions include the severity of: hyperactivity/aggression, self-injury, alcohol or drug problems, cognitive problems, physical problems, psychotic complaints, depressive mood, ‘other’ psychological and behavioral problems, problems with social relationships, daily functioning, housing conditions, and daily activities. It takes 5–10 minutes for a practitioner to complete the HoNOS. Item scores range from 0 (no problem) to 4 (very serious problem). The subscores of the 12 questions can be added for the total sumscore (range 0–48). There are reference groups with SMI patients available. Psychometric properties of the HoNOS are satisfactory to good [30]. The total sumscore as well as the score on all individual questions (subscores) were used to identify possible associations with a high risk of SUD.

### Manchester Short Assessment of quality of life (MANSA)

The MANSA is a multi-dimensional self-rating questionnaire about quality of life as experienced by the patient [31]. It consists of 12 items about satisfaction with various domains of life: life in general, house, roommates, occupation, physical and mental health, social and family relationship, current relationship, personal safety, sexual life and financial situation. The satisfaction is scored on a seven-point rating scale (1 = extremely negative, 7 = extremely positive), which leads to MANSA-subscores for each question separately. There are also four yes/no questions about friendship and safety (if a person has friends, if he has spoken one the last week, if the person was victim of violence, and if the person was accused of crime in the last year). The psychometric properties of the MANSA are satisfactory [33]. In the present study, the summed scores (range 12–84), as well as the item scores of all domains and the yes/no questions, were used to identify possible associations with a high risk of SUD.

### DSM-5 substance use disorder classifications

The clinically obtained DSM-5 SUD classification with the date the closest to the date of the TAPS, was gathered from the patient files. In the Netherlands, these are classified by a psychologist or psychiatrist.

### Statistical analyses

#### Prevalence

The frequency of used substances according to the TAPS 1 and TAPS 2 were calculated. We computed percentages of high risk of SUD (a score of +2 on any of the categories of the TAPS-2) per substance category.

#### Under-detection

To determine possible underdetection of SUD, we compared the percentage of a high risk of SUD according the TAPS-2 to the percentage of patients who had a clinically obtained DSM-5 classification of SUD. We investigated the percentage agreement of the TAPS outcome (low/high risk of SUD) with the DSM-5 classification (presence or absence of a SUD) and tested the significance of this association by Cramer’s V and Cohen’s Kappa. We also investigated the correspondence between the type of SUD (alcohol, drugs, medication) in the DSM-5 classification and type of substance with a high risk of SUD on the TAPS-2.

#### Associations

We explored the predictive value of patient characteristics on a high risk of a SUD (TAPS-2 score ≥ 2) with a multivariate logistic regression analysis. The investigated variables were: satisfaction of life (MANSA total score and subscores), level of education, main DSM-5 classification, gender, partner, ethnicity, mental and social functioning (HoNOS total score and subscores) and overall functioning (GAF score). The procedure was as follows: first, we investigated if the variables of interest had enough relevance to be added to the logistic regression analysis. We used the relevance criterion of Hosmer and Lemeshow: variables in which the association had a *p*-value < .2 were included in the analysis [34]. To determine this, we used a chi-square test for dichotomous variables and a *t*-test for independent samples for continuous variables. Next to this, we put the relevant variables one by one in a multivariate logistic regression analysis through the forward entry backward correction method. We did this in two steps to deal with the small number of cases in relationship to the number of predictors. First, we only included demographic variables, inspecting McFadden’s R square. Then we added variables from the MANSA to the model, again inspecting McFadden’s R square. Finally, we included variables with a significant contribution in the first two steps into the final model. A McFadden’s R square of < .2 is low, between .2 and .4 is reasonable, and > .4 good [35].

## Results

### Sample characteristics

Characteristics of the 83 patients are described in Table 1. There were slightly more men. One-fifth had a partner. One-sixth were from an ethnic minority. Almost two-third of the sample had a middle or high education. A psychotic disorder was the most common disorder. Two fifth were classified with a DSM-5 SUD next to the main classified disorder, a quarter of which also had a second SUD classified disorder.

**Table 1:** Participant characteristics (N = 83).

Age	
Mean (*SD*)	42.7 (12.0)
Range	22–68 years
Gender (%)	
Male	55
Female	45
With partner (%)	21
Ethnic minority (%)	16
Main classified disorder DSM-5 (%)	
Psychotic disorder	47
Anxiety/mood disorder	27
Developmental disorder (autism, ADHD)	8
Personality disorder	16
Classified SUD DSM-5 in patient file (%)	39
Alcohol	16
Cannabis	12
Amphetamine	8
Medication (sedatives or opioids)	5
Caffeine	5
Nicotine	1
Cocaine	1
Second SUD classification (%)	10
Education (%)	
No education / special education	11
Low	25
Middle / average	48
High	16

### Prevalence—TAPS 1 and 2 outcomes

The rate of substance use and high risk of SUD is presented in Table 2 for TAPS-1 and Table 3 for TAPS-2 outcomes. TAPS-1 shows the past-year use of the four categories (tobacco, above guideline-recommended daily limits of alcohol, illicit drugs and prescription medications for nonmedical purposes) for all patients. These results show that 89% of the patients used one or more of the four categories of substances: three-quarter smoked tobacco of which more than 90% on a daily basis, around two-third consumed above guideline-recommended daily limits of alcohol, almost half used illicit drugs and a quarter reported the use of non-prescribed medication. One-third of these patients used two or three categories, one-fifth used one category and 10% used all four categories.

**Table 2:** TAPS-1 outcomes–prevalence and frequency of use.

N = 83	Tobacco	Alcohol	Drugs	Prescription medication
No use (%)	24	37	57	77
Any use (%)	76	63	43	23
Frequency (%)				
• Less than monthly	6	37	28	47
• Monthly	2	17	11	32
• Weekly	0	37	19	11
• (Almost) daily	92	10	42	11

**Table 3:** TAPS-2 outcomes–prevalence and high risk of SUD.

*N* = 83	*Tobacco*	*Alcohol*	*Marihuana*	*Stimulant drugs*	*Heroin*	*Opioids*	*Sedatives*	*Stimulant*
								*Medication*
No use (%)	29	42	68	87	99	99	89	98
Use (% and number of patients)	71	58	32	13	1	1	11	2
	N = 59	N = 48	N = 27	N = 11	N = 1	N = 1	N = 9	N = 2
High risk SUD (%)								
	68*	31*	24*	10*	1*	1*	7*	2*
	95**	54**	74**	73**	100**	100**	67**	100**

Note

* = percentage of the total sample (*N* = 83)

** = percentage of the users

The number of patients of the TAPS-2 was slightly different than just the users of TAPS-1 because a part of the patients used one of the categories in the last 12 months, but not the last three months. TAPS-2 results showed that almost all smokers, half of the alcohol users and three-quarter of the patients that used marihuana or stimulant drugs had a high risk of SUD according to the TAPS-2. Marihuana was the most commonly used drug followed by stimulant drugs. Only one patient used heroine. Most patients who misused medication used sedatives for this purpose. A high risk of SUD because of medication misuse was rare in this sample (1–7%). Looking at combinations of psychoactive substances used (not listed in the table), the main finding is that patients at high risk of alcohol, drugs or prescription medication-misuse related SUD also have a tobacco use disorder.

### Under-detection

A high risk of SUD is far more present than the SUD classification in the patient file indicates. Before start of data collection, 39% had a SUD classification in their patient file. After taking the TAPS, it was found that 81% were at high risk of SUD. The percentage agreement between the TAPS-2 outcome (high / low risk of SUD) and the classification of SUD in the patient (yes / no) file was low (53%), as was the Cohen’s Kappa with 0.176. 94% of the patients with a SUD in their file had a TAPS-2 score that indicates they were at high risk of SUD. However, 73% of the patients who had no SUD classification in the patient file, the TAPS-2 outcome showed to be at high risk of SUD. In fact, the difference between a high risk of SUD according the TAPS-2 and a SUD classification in the patient file was significant (Chi-square = 5.68; Fisher’s exact test *p* = .022). The strength of the relationship between the TAPS-2 outcome and the DSM-5 classification was medium (Cramer’s V = 0.262).

Secondary analysis:

Because research on addiction in patients with SMI mostly focusses on other addictions than tobacco [11, 12], we did a secondary analysis without the patients that only had a high risk of SUD because of tobacco use. In this smaller group (*N* = 60), 42% had a SUD in their patient file, whereas 73% had a risk of SUD according the TAPS-2. Sixty percent of the patients without a SUD classification in their patient file scored high risk for SUD on the TAPS-2. The percentage agreement of this smaller sample (*N* = 60) rose to 62% compared to the percentage agreement of the total sample (*N* = 83). The under detection of SUD in this smaller sample—without SUD only because of tobacco use—remained significant and was even more powerful (Fisher’s exact test *p =* .007). The strength of the relationship increased a little (Cramer’s V = .357).

In the total sample (*N* = 83), for 32% of the patients the DSM-5 classified disorder corresponded with the TAPS-2 outcome. For example, a DSM-5 SUD on alcohol corresponded with a high risk of SUD of alcohol problems on the TAPS-2. Of the 68% of the patients at high risk of problematic tobacco use (*N* = 40) only one patient had a SUD for nicotine in the DSM-5 classification (note that the TAPS asks for tobacco use and the DSM 5 classifies nicotine SUD). Half of the patients at high risk of SUD on alcohol had a DSM-5 classification SUD on alcohol. Almost 60% at high risk of SUD on drugs had a DSM-5 SUD classification on drugs. For medication, there was no association: none of the patients with a risk of SUD on medication according the TAPS had an according DSM-5 SUD classification and none of the patients with a DSM-5 SUD classification on medication had an elevated score on the TAPS-2.

### Associations

Table 4 presents associations between ‘at high risk of SUD’ according the TAPS-2 (score ≥ 2) and several independent variables. Associations were with: gender (SUD in male = 89%, in female = 70%; Cramers V = 0.238; Fisher’s exact test = .048), having a partner (having no partner and SUD = 84.8%, having a partner and SUD = 64.7%; Cramers V = 0.206; Fisher’s exact test = .084), ethnic minority (no association; Fisher’s exact test .446), having a SUD classification DSM-5 (No SUD classified but SUD according to TAPS = 72.3%; a diagnosis SUD and in TAPS = 93.8%; Chi-square *p* = .017), age 48 in patients with and 41 in those without SUD; *t*-test *p* = .040), MANSA satisfaction about the relationship with family (Mean with SUD = 5.37; without SUD = 4.37; *t*-test *p* = .031; effect size = 0.571), MANSA satisfaction with the financial situation (Mean with SUD = 5.01; without SUD = 4.10; *t*-test *p* = .073; Effect size = 0.519) and HoNOS score on alcohol and drug abuse (Mean with SUD = 1.34; without 0.44; *t*-test *p* < .001, Effect size = -0.767). The univariate regression analysis with the relevant variables showed that a low level of education had no significant association with TAPS-2 score ≥ 2. This variable was left out of the multivariate regression analysis. HoNOS alcohol and drug abuse and SUD classification DSM-5 were not included because of collinearity with the outcome variable.

**Table 4:** Predictive value of patient characteristics, HoNOS, MANSA for a high risk of SUD (TAPS-2).

	*High risk of SUD (TAPS 2 ≥ 2)*						
	*Univariable association*			*Multivariable association*			
*Variable*	*Ex (b)*	*p*	*95% CI Ex (b)*	*B*	*Ex (b)*	*p*	*95% CI Ex (b)*
Partner	3.055	.068	[.919–10.151]				
SUD classification DSM-5	5.676	.029	[1.195–26.952]				
Male gender	3.469	.036	[1.081–11.130]	1.608	4.994	.023	[1.242–20.076]
No partner	.327	.068	[.099–1.088]	1.142	3.133	.103	[.794–12.356]
Age	.952	.046	[.906 - .999]	-.076	.927	.018	[.870 - .987]
MANSA satisfaction with financial situation	.735	.080	[.520–1.038]				
MANSA satisfaction about relationship with family	.704	.058	[.490–1.012]	-.491	.612	.022	[.403 - .931]
HoNOS alcohol and drug abuse	.456	.014	[.244 - .853]				

After backward deselection, male gender, having a partner, age and MANSA satisfaction about the relationship with family showed a significant association with a TAPS-2 score ≥ 2 in the multivariate regression. Males were five times more likely to have a risk of SUD, and lack of a partner made it three times more likely to have a risk of SUD. With each year that age increases, the risk of a SUD drops with almost 5%. More satisfaction about the relationship with family lowered the risk of a SUD. The McFadden’s R square of the demographic variables was .147. The Mc Fadden’s R square of the MANSA items was. 087. The McFadden’s R square of the final model was 0.248. In short, we may estimate an explained variance of approximately 25% which is reasonably good with 83 cases and five predictors.

Secondary analysis including only the patients (*N* = 60) with a high risk of SUD (TAPS-2 score ≥ 2) because of alcohol, drugs or medication showed that tobacco use made it 58 times more likely to have a high risk of SUD on one of the other categories (univariate regression analysis: Exp(B) = 58.333; *p* < .001).

## Discussion

Our results show a high prevalence of substance use among SMI patients. At least half of the patients with substance use also were at high risk of SUD according to the TAPS. TAPS outcomes compared to the DSM-5 classification reveal a great amount of under-detection of substance use problems. Gender, partnership, age and satisfaction about the relationship with family are associated with a high risk of SUD. We discuss these outcomes below.

### Prevalence

Our results revealed a high co-occurrence of used substances use in a SMI sample receiving F-ACT: 90% used at least one of the following four categories in the past year: tobacco, above guideline-recommended daily limits of alcohol, drugs or non-prescribed medication. For 80% there was a high risk of SUD according to the TAPS-2, which means actual use and presence of at least two features of SUD. These prevalences are higher than those mentioned in other research [8, 10]. This could be due to the inclusion of tobacco dependency in the TAPS (excluded in other prevalence studies). In line with [11, 12, 14] we also found that, besides tobacco, alcohol is the most used substance, followed by marihuana.

Both the prevalence of use of tobacco (76%) and being at high risk of a SUD because of tobacco use (72%) in this SMI sample were much higher than the national Dutch population average (i.e., 19%) (retrieved from www.cbs.nl). This is in line with findings in the United States, United Kingdom and Australia also showing an association of the prevalence of smoking and the number of mental disorders: 18% of the people without a mental illness to 61% of the people diagnosed with 3 or more mental disorders smoke [36].

The risk of nicotine use is particularly neglected in the literature while it is of great importance when it comes to the morbidity and mortality of patients with SMI. Among people with schizophrenia, of patients who have a 12–15 years shorter life expectancy than the general population, more than two-thirds die of coronary heart disease [37]. Twice as many patients with SMI die as a result of cardiovascular problems compared to people from the general population. Smoking is the most important risk factor for poor cardiovascular outcomes and is an even larger risk for cardiovascular problems than hypertension alone, diabetes, dyslipidemia, metabolic syndrome, obesity and obstructive sleep apnea [38]. However, this also raises ethical questions. Especially for the elderly, who grew up in a time when smoking was very common, one might ask whether it is right to ask these vulnerable people to remove their ’pleasure’ in smoking. Our results do show that smoking is an important predictor for the development of other addictions, as smoking is 58 times more likely to be at high risk of SUD than any other category. This makes it even more important to understand smoking behavior and find a balance in whether or not to motivate smoking cessation.

### Under-detection

The TAPS clearly showed a higher risk of SUD than the clinicians’ DSM-5 classification. This can be due to a difference in the items in the TAPS versus criteria for the DSM-5. The TAPS-tool highlights important but not all DSM-5 symptoms for SUD. Another reason can be the difference in reference person: the DSM-5 classification is the opinion of the clinician, and the TAPS is self-administered. Another possibility is that SUD classifications are only made by clinicians if during treatment moderate or severe addiction problems are likely. A complicating factor in a thorough assessment of substance use problems, whether with a screener or in a conversation with the patient, is the reluctance in sharing this information. The main focus of treatment for patients with SMI in F-ACT is on the burden of their complex psychopathology other than the addiction. Patients may hold back information about used substances or features of addiction because of the fear of possible negative consequences like getting no medication or exclusion from treatment. This seems to be especially the case for misuse of medication due to dependence on the F-ACT psychiatrist who prescribes their medication. Another explanation for the difference between TAPS and the DSM-5 classification is that the TAPS is only a screener and brief assessment. A DSM-5 clinical interview is the comprehensive assessment for correctly classifying an SUD. It may be that, if after finding a positive result on the TAPS, a re-interview according to DSM-5 for SUD might find a higher rate of SUD than during a clinical interview at intake without the TAPS screen administered first. Also, the TAPS is a screener so it will find more at risk cases that perhaps might be sub-threshold SUD. In a SMI population, it would be helpful to know about substance use and consequent problems even if they don’t reach the SUD threshold because substance use can mimic many mental health symptoms such as depression, mania, or a personality disorder. Their use can also exacerbate symptoms and occurring use is likely associated with suicide [13, 15, 16]. The worsening of psychiatric symptoms due to substance use may also be the reason why significant others are concerned about a patient’s substance use.

### Associations

Gender, age, partnership and relation with family were associated with being at high risk of SUD according to the TAPS. Considering gender, studies have shown that males report greater substance use than women: men drink more alcohol, use more stimulant drugs, are more likely to use cannabis and start using it at a younger age, use more tobacco, have more frequent heroin use and use greater quantities than women [39]. Our results are in line with this. Gender differences vary among the different stages of addiction such as acquisition, escalation, maintenance, withdrawal and relapse due to biological, psychological and social differences between men and women [40]. Longitudinal studies on alcohol and drugs problems confirm our results regarding the decrease of SUD with increasing age. SUD frequency increases during adolescence and early adulthood, peak in the early twenties and decrease afterwards, also known as ‘maturing out’ [41].

The importance of social support and qualitatively good relationships with others is widespread in the literature. A meta-analysis of 131 longitudinal studies supports the importance of good social relations [42]. Having a limited supportive social network increases the risk of SUD. Being an outsider increases the risk of SUD on the other hand [43]. Thus, our findings on variables associated with a high risk of SUD in our study are in line with previous findings.

### Strengths and limitations

A strength of this study is that the results are obtained in a clinical setting in which the used instruments are part of standard care. Therefore, the results are a good representation of the situation in the clinical practice of SMI patients in F-ACT treatment. This highlights the clinical value of a short screener like the TAPS in a complex patient population with multiple mental health complaints and psychosocial problems.

The TAPS-tool is available in English, Spanish and Dutch. Psychometric properties are investigated in a large sample of primary care patients (*N* = 2000, [18]) and pharmacy patients (*N* = 1523, [25]). A limitation of this study is that we did not examine psychometric properties in this relatively small Dutch SMI sample. The vulnerability and the limited taxability of the patient group makes a psychometric evaluation with an extensive reference instrument difficult to achieve. However, the results in terms of prevalence, under-detection and associations seemed so important to us that we felt they should be reported. Another weakness is the limited generalizability due to the small sample size. Despite the exclusion of patients with MID, there were difficulties with some of the relatively long sentences and to some extend difficult wording of the items of the TAPS-2. This is also the reason that we are developing a version adapted to patients with MID or lower intelligence. Finally, the TAPS does not include addictions such as gaming, eating, buying, new psychoactive substance and sex and love addiction.

### Implications for clinical practice

The TAPS-tool seems an appropriate screening instrument in this population because of its short and easy administration and it can be used as self-report questionnaire or interview. The short duration makes it also feasible for patients with cognitive or attentional problems. A high risk of SUD on the TAPS must be followed by a broader assessment, which includes gathering information about consumption patterns, substance-related life problems, expectancies, situational contexts, impact on psychiatric symptoms and motives for use. Assessment leads to a substance-use related diagnosis according to the DSM-5 criteria and indication for treatment and evaluation of its effectiveness. For the best detection, one needs to combine several sources at the beginning of treatment, and include follow-up sessions in the treatment. Various sources can be: self-report questionnaires, blood/urine tests, asking the social support system and watching for signs in the home situation. This could be done in FACT-teams by case managers and home visit.

F-ACT treatment focuses on all biopsychosocial domains of recovery, such as investment in social support and social relations. As a result, these teams may be ideally suited for screening for SUD and help SMI patients get the care they need. A review [44] confirms positive effects of assertive community treatment for patients with SMI and substance use. This includes lower prevalence and severity of alcohol and drug use, increased stage of change in substance use treatment, and fewer days of hospitalization and intoxication. Collaborations with general practitioners and addiction services are needed to provide the best treatment options. Preferably through integrated instead of parallel treatment.

## Conclusion

Screening for addiction in a SMI sample with the TAPS-tool reveals a large number of patients with a high risk of SUD, substantial under-detection, and associations of male gender, partnership, age and satisfaction about the relationship with family with a high risk of SUD. Tobacco addiction in particular requires more attention in treatment to provide health care benefits. Our study underlines the importance of assessment of substance use and misuse at the start of treatment and during annually evaluations.

## Supporting information

S1 Fig TAPS 1 and 2: Items, skip patterns and scoring. (DOCX)

S1 Data (XLSX)

S2 Data (XLSX)
