# Supplementary material for: TAPS-tool reveals severe under detection of substance use problems in patients with severe mental illness
Source: PLoS One. 2024 Jul 24;19(7):e0305142. doi: 10.1371/journal.pone.0305142 (PMC11268639; doi:10.1371/journal.pone.0305142)
Supplement: S1 Fig — (DOCX) [file pone.0305142.s001.docx]

**S1 Fig: TAPS 1 and 2: items, skip patterns and scoring**

*
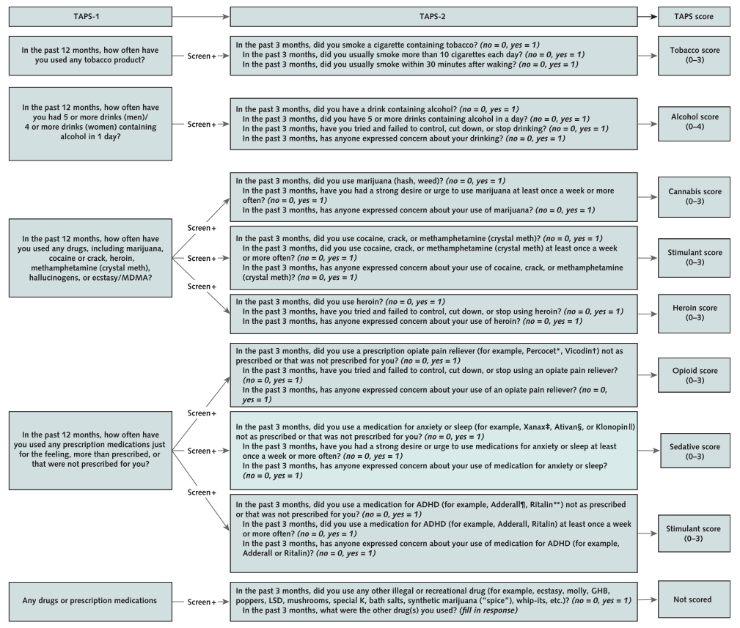
*

From: *Annals of Internal Medicine*, McNeely J, Wu LT, Subramaniam G, Sharma G, Cathers LA, Svikis D, et al. Performance of the Tobacco, Alcohol, Prescription Medication, and Other Substance Use (TAPS) Tool for Substance Use Screening in Primary Care Patients. 2016;165(10):690-9. doi:10.7326/M16-0317.

Copyright © [2023] American College of Physicians. All Rights Reserved. Reprinted with the permission of American College of Physicians, Inc.
